# Feasibility of Therapist‐Driven MR‐Guided Adaptive Radiotherapy for Oligometastatic Disease: Geometric Accuracy and Dosimetric Impact

**DOI:** 10.1111/1754-9485.70016

**Published:** 2025-08-26

**Authors:** Amanda Moreira, Winnie Li, Iymad R. Mansour, Mame Faye, Ali Hosni, Aruz Mesci, Enrique Gutierrez‐Valencia, Patricia Lindsay, Peter Chung, Jeff Winter

**Affiliations:** ^1^ Radiation Medicine Program, Princess Margaret Cancer Centre Toronto Ontario Canada; ^2^ Department of Radiation Oncology University of Toronto Toronto Ontario Canada

**Keywords:** adaptive radiotherapy, contouring, dosimetric impact, MR‐Linac, oligometastatic disease, RT‐led workflow

## Abstract

**Introduction:**

MRI‐guided adaptive radiation therapy (ART) is the resource‐intensive process of daily treatment plan modification. This study aims to demonstrate the feasibility of radiation therapist (RT)‐led MRI‐guided ART for oligometastatic disease (OMD) by comparing geometric accuracy and dosimetric differences between RT and radiation oncologist (RO) re‐contouring.

**Methods:**

Five RTs and five ROs retrospectively re‐contoured gross target volumes (GTVs) and organs‐at‐risk (OARs) for eight OMD cases. RT and RO contours were compared against consensus RO Simultaneous Truth and Performance Level Estimation (RO‐STAPLE) contours using the Dice similarity coefficient (DICE), mean distance to agreement (MDA), planning target volume (PTV) D95 and OAR D0.5cc using the Wilcoxon signed‐rank test. Moreover, an RO qualitatively scored all contours using a 5‐point Likert scale.

**Results:**

We found very good geometric accuracy with average (±standard deviation) GTV DICE of 0.82 ± 0.06 for RTs and 0.85 ± 0.09 for ROs and MDA of 0.88 ± 0.03 mm for RT and 0.75 ± 0.05 mm for ROs relative to the RO‐STAPLE. Qualitative GTV Likert scores were excellent, 4.8/5 for RTs and 4.7/5 for ROs. Mean percent difference in PTV D95 compared to RO‐STAPLE was small but significantly higher for RTs (0.5% ± 1.5%) compared with ROs (−0.7% ± 1.9%, *p* < 0.05). Mean relative change in OAR D0.5cc results was small with −1% ± 6% for RTs and −1% ± 12% for ROs.

**Conclusions:**

Here we provide the first report of geometric and dosimetric contouring uncertainty for MR‐guided online ART for OMD. Our results show that RT re‐contouring maintains similar performance for eligible targets and OARs compared with RO contours, establishing the initial feasibility of an RT‐led workflow.

## 1 Introduction

Treatment of oligometastatic disease (OMD) with local ablative radiotherapy relies on advanced technology and techniques to pursue curative intent [1]. Integrated MRI linear accelerators (MRL) have led to MR‐guided radiotherapy, which leverages the improved MRI soft tissue contrast to visualise and delineate gross tumour volumes (GTVs) and organs‐at‐risk (OARs) in OMD [2]. Additionally, the ability to account for daily variation in target and OAR structures, particularly luminal structures, is beneficial for the treatment of OMD [3]. MR‐guided online‐adaptive radiotherapy (ART) allows safe and efficacious target dose delivery while simultaneously sparing OARs based on the anatomy of the day [4, 5].

One drawback of MR‐guided daily ART is that target and OAR contour revision is time consuming and resource intensive as it requires contouring expertise, which is typically achieved by having a radiation oncologist (RO) present at the treatment unit for every fraction. Shifting responsibility for contouring to the treatment radiation therapists (RTs) already at the unit supporting treatment delivery will remove the need for scheduling additional staff with contouring expertise and help improve utilisation of MR‐guided ART for OMD. For prostate treatment on the MRL, multiple studies have reported on the accuracy and efficacy of radiation oncologist delegating the online contouring and plan re‐optimisation role and responsibilities to well‐trained RTs [6, 7, 8]. These reports of RT‐led MR‐guided ART demonstrate that with appropriate training it is possible for RTs to support online contouring. As with primary prostate cancer, oligometastatic treatments might benefit from increased patient throughput and access offered by RT‐led MR‐guided ART [9, 10]. Introducing an RT‐led MR‐guided OMD workflow is hampered by the paucity of inter‐observer MRI contour variability data for targets and relevant OARs. Moreover, there is no data directly comparing RT and RO contouring accuracy for OMD in a realistic time‐constrained ART workflow.

Here we describe a training programme developed for RTs to support online adaptive MR‐guided OMD contouring and the qualitative and quantitative contouring accuracy evaluation for the first RT cohort. Given the limited data evaluating inter‐observer contouring variability for MR‐guided ART in OMD, we performed a comprehensive evaluation of geometric accuracy, dosimetric uncertainty and qualitative performance of RT contouring compared with ROs in a simulated daily ART workflow, using a consensus RO‐contour as the gold standard for comparison for a cohort of cases deemed eligible for a RT‐led workflow. This study aimed to demonstrate the feasibility of RT‐led MRI‐guided ART for OMD by comparing geometric accuracy and dosimetric differences between RT and RO re‐contouring.

## 2 Methods

This project was reviewed and approved by our institutional quality improvement review committee, University Health Network's Quality Improvement Review Committee (ID #24‐0793). All RTs taking part in this evaluation previously completed our in‐house developed 3‐phase training program for RT‐led online ART for prostate cancer, which established a competency framework for contouring prostate, bladder, rectum and bowels [8]. This pilot evaluation of OMD patients represents the first of three phases in a process mirroring the previously published prostate training program for a RT‐led workflow [8].

### 2.1 Patient Population

**FIGURE 1:**
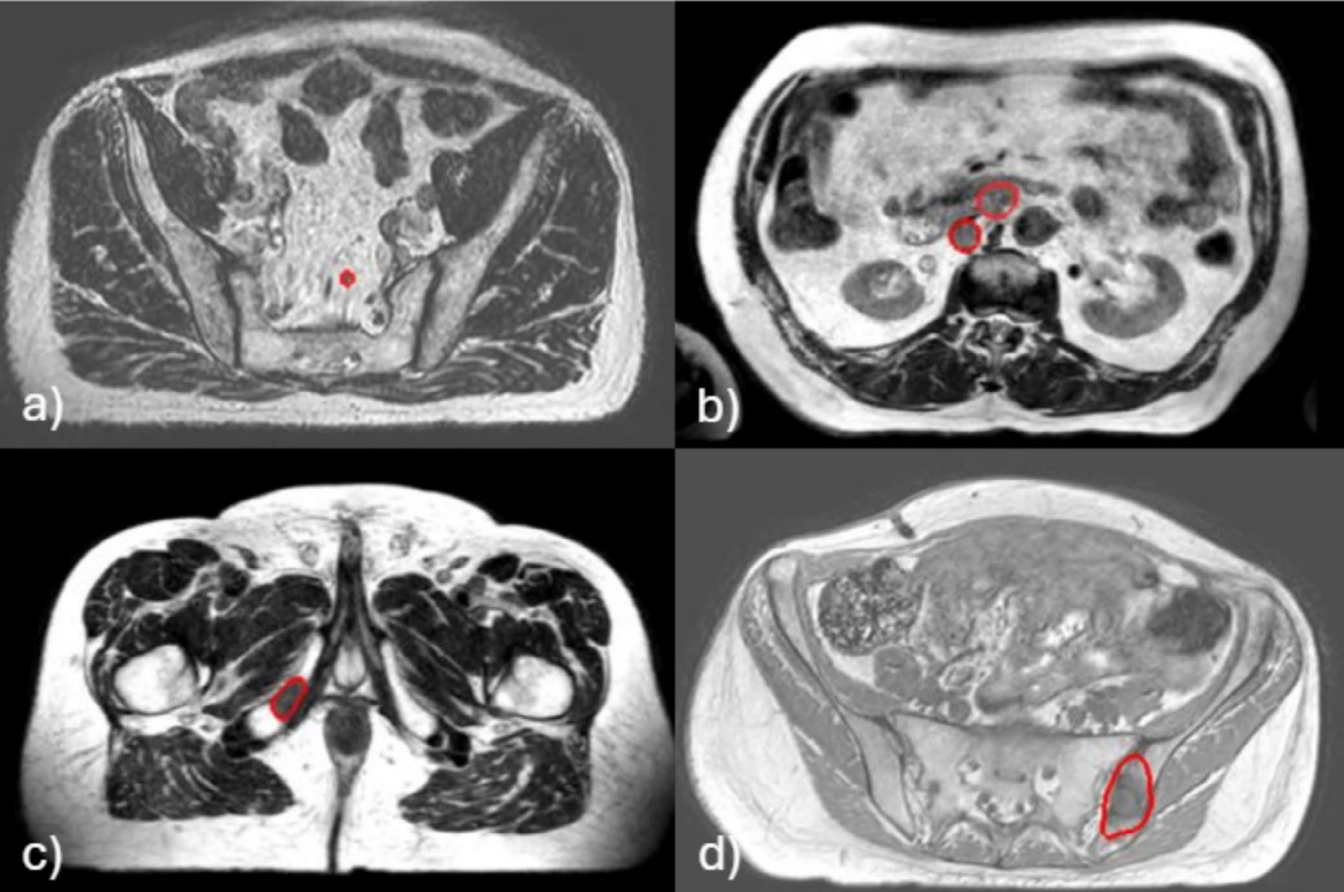
Examples of eligible oligometastatic targets by classification, (a) soft tissue—simple, (b) soft tissue—complex, (c) bone—simple and (d) bone—complex.

For this pilot evaluation, we selected cases representative of our OMD referral patterns and considered metastatic cases with high expected variability over their treatment course, such as those in the liver and pancreas, to be ineligible. We defined eligibility for RT‐led workflow as extra‐hepatic/non‐pancreas soft tissue or bone metastases given the high target contrast within pelvic/abdominal fat and limited target motion. These eligible cases comprise approximately 65% of all oligometastatic patients treated on the MRL (Figure S1). Of the 41 extra‐hepatic/non‐pancreas cases treated between January 2021 and December 2023, cases were categorised by subtype (soft tissue vs. bone metastases) and then complexity. Simple cases had distinct target borders and minimal bowel and stomach variation relative to simulation imaging (e.g., single bone metastasis). Eligible complex cases had targets with diffuse borders, multiple targets and large OAR change relative to simulation imaging (e.g., gastro‐hepatic lymph node) (Figure 1). Lists of OARs found within 2 cm of the targets were created and cases containing the proximal OARs identified for evaluation were selected, while simultaneously ensuring a mix of simple and complex case representation (see Figure S2). We selected 5 soft tissue and 3 bone metastases, classified as simple (soft tissue *n* = 3 and bone *n* = 2) and complex (soft tissue *n* = 2 and bone *n* = 1).

### 2.2 RT Training for OMD

To translate our RT‐led prostate primary contouring to OMD, we identified the following additional needs: OMD GTV contouring, additional pelvic OAR contouring (e.g., sacral plexus, ureters) and abdominal OARs (e.g., stomach, duodenum). We asked all RT participants to indicate their years of clinical experience, MRL experience and to rate self‐reported competencies in various ART activities.

In April 2024, the training program started with a site‐specific 1‐h anatomy review workshop for all RT participants, including MRI appearance of radiotherapy targets (bone metastasis and pelvic/abdominal lymph nodes), origins and innervations of the lumbosacral plexus and sciatica nerve, and normal MRI appearance of both pelvic and abdominal luminal structures (e.g., large bowel, small bowel, GE junction). The session also identified typical post‐surgical anatomical variations such as bowel definition post Whipple or lymphocele identification. Following the workshop, the next phase was contour generation described below.

### 2.3 Contour Generation

We retrieved and anonymised training MRIs from eight previously treated MRL patients fulfilling our eligibility criteria with OMD treated on an IRB‐approved study (ID 19‐5843) in the MR‐Linac treatment planning system (Monaco, Elekta AB, Stockholm, Sweden). Following the anatomy review session, 5 RTs were asked to register the adaptive session MR to the reference MR which resulted in the target's rigid propagation and OAR's deformable propagation onto the adaptive session MR. RTs then re‐contoured both target volumes and all OARs within a region of 2 cm from the PTV. A 15‐min time limit per case, including registration and re‐contouring, simulated our time‐sensitive online workflow that minimises treatment time and intrafraction motion. Five ROs completed the same exercise to evaluate RT contouring performance relative to ROs, establish baseline RO inter‐observer contouring variability and generate consensus gold standard contours.

### 2.4 Quantitative and Qualitative Assessment

We generated Simultaneous Truth and Performance Level Estimation (STAPLE) [11] consensus contours for GTVs and relevant OAR contours for the 5 participating ROs (RO‐STAPLE) and RTs (RT‐STAPLE). Briefly, a STAPLE contour estimates the underlying ground truth contour from a collection of contours, in which each contour independently approximates the underlying structure using an expectation‐maximised approach to create a STAPLE contour via pixelwise classification of the input contour set [12]. For this study, STAPLE contours were calculated using the scikit‐rt library with the get_staple function with default control parameters [13]. We compared RO‐STAPLE versus RT‐STAPLE contours to assess systematic inter‐profession consensus contouring differences as well as compared each RT and RO contour versus the RO‐STAPLE contour using the DICE similarity coefficient, mean and maximum distance to agreement (DTA) [14].

One RO who did not participate in the contouring exercise also performed a qualitative contouring assessment blinded to the professional groupings using a 5‐point Likert scale (1 = ‘very poor’ and 5 = ‘excellent’). Each case was assigned a mark for the GTV contour and the collective OAR contours. A Likert score of ≥ 4 was deemed a ‘pass’, and for credentialing of an RT‐led workflow, the desired pass rate for each RT was set at 90% of cases. Any RTs who did not receive a ‘pass’ had an additional feedback session with the RO and completed 5 additional contouring cases.

To capture dosimetric impact of contour variation, we extracted PTV and relevant OAR dose‐volume metrics for the contours using the online re‐optimised adapted plan. Our PTV margin was a uniform 5 mm GTV expansion for all cases except Cases 2 and 7 which were treated after a departmental shift to a uniform 4 mm expansion. We extracted the RT, RO and RO‐STAPLE PTV and OAR doses using the RayStation v10B treatment planning system (RaySearch Laboratories, Stockholm, Sweden). For PTV dose metric differences we assessed the dose to 95% of the volume (D95) normalised to the prescription dose to allow comparison of different dose fractionations. Variation in PTV D95 was defined as ‘acceptable’ for changes < 1%, ‘minor deviation’ for changes between 1% and 3%, ‘clinically relevant deviations’ for changes between 3% and 5% and ‘major deviation’ for changes > 5%. For relative dose differences to critical OARs, we evaluated dose to 0.5 cc volume (D0.5cc) for luminal (e.g., bowel) and nerve (e.g., sacral plexus). For evaluation the large and small bowels were combined in a single ‘bowel’ contour. All dosimetric comparisons were made between RT and RO contours relative to the RO‐STAPLE contour using the clinically treated plan. To support use of the clinically treated plan for this contour comparison, we performed a preliminary assessment to ensure minimal differences existed between the online adapted PTV and RO‐STAPLE PTV contours.

### 2.5 Statistical Analysis

We used descriptive statistics to report geometric and dosimetric differences between professions and OMD target types. We compared RT and RO contour differences from the RO‐STAPLE using the Wilcoxon signed rank test with *p* < 0.05 considered statistically significant following the Benjamini–Hochberg correction for multiple comparisons. All box‐and‐whisker plots represent the median (solid line), inter‐quartile range (IQR; box) and the ‘x’ indicating the mean value in each group.

## 3 Results

All RTs reported at least 5 years of general practice, with all but 1 having at least 2 years of experience in the MRL (Figure S3). Most RTs rated their competency in various ART tasks as ‘competent’ or higher. All ‘Novice’ or ‘Beginner’ responses came from a single RT with < 2 years MRL experience. ROs who completed the contouring exercise all worked in the MRL, were responsible for contours during online ART sessions, and included fellows and staff ROs.

**FIGURE 2:**
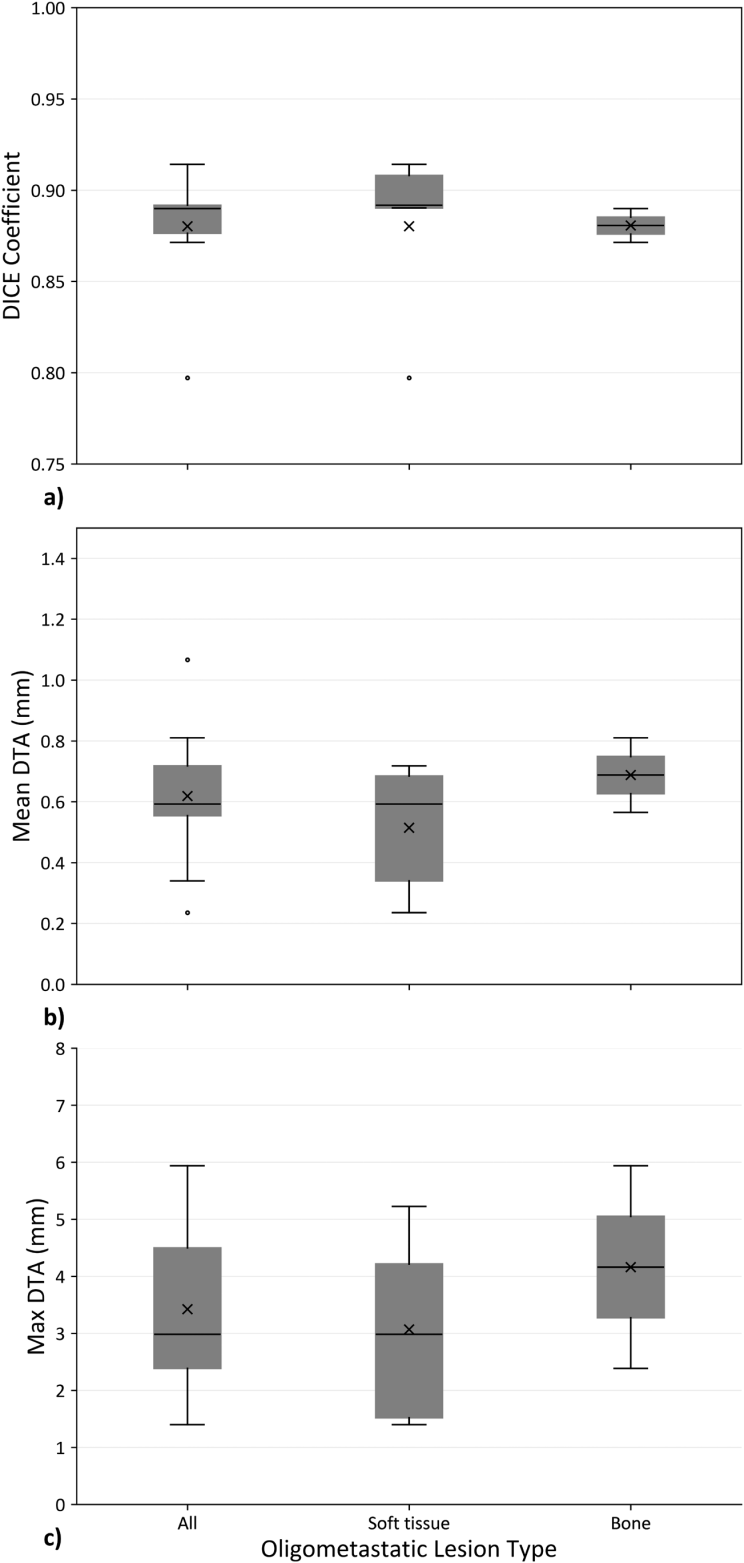
Geometric comparison of the eligible GTV consensus contours for radiation therapist (RT) and radiation oncologist (RO) consensus contours generated using the Simultaneous Truth and Performance Level Estimation STAPLE. Comparisons include (a) DICE coefficient, (b) mean distance to agreement (DTA) and (c) maximum DTA contours for different oligometastatic target types. The ‘x’ represents the mean value in the plot.

**FIGURE 3:**
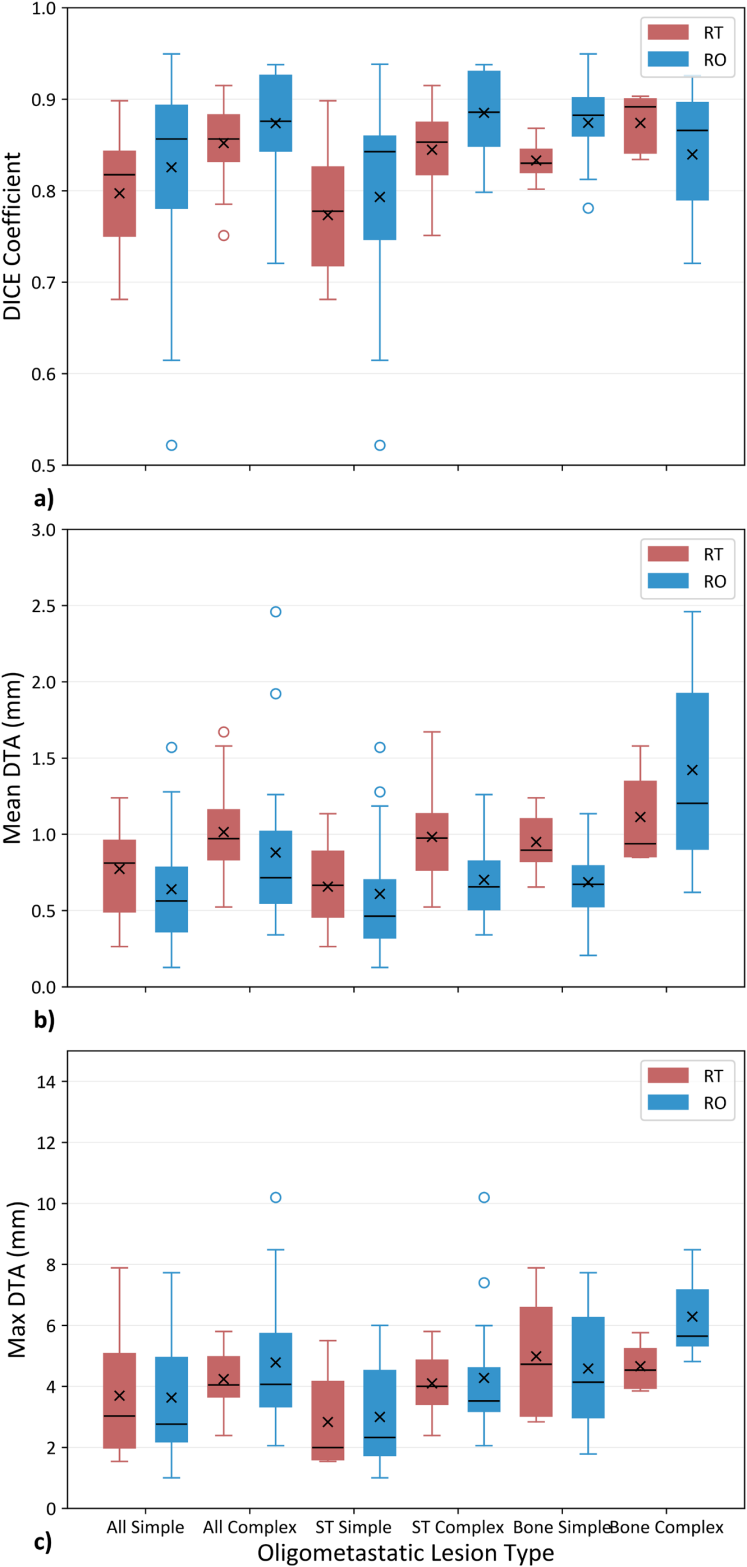
Target GTV volume DICE scores (a), mean distance to agreement (mean DTA; b) and maximum DTA (max DTA; c) by complexity for both professional cohorts versus RO consensus contour with ‘x’ illustrating the mean value.

Geometrically comparing RT‐STAPLE to RO‐STAPLE, averaged for all 9 targets, the DICE (±standard deviation) was 0.88 ± 0.03, mean/max DTA was 0.6 ± 0.2/3.4 ± 1.6 mm (Figure 2). Comparing individual RT and RO GTV contours versus the RO‐STAPLE, the mean DICE was 0.82 ± 0.06 for RTs and 0.85 ± 0.09 for ROs, with variations in DICE scores across patient subgroups shown in Figure 3. Averaged across all eligible GTV subtypes, the mean/max DTA was 0.88 ± 0.03/3.9 ± 0.2 mm for RT and 0.75 ± 0.05/4.1 ± 0.2 mm for RO contouring compared to the RO‐STAPLE, with no differences reaching statistical significance.

**FIGURE 4:**
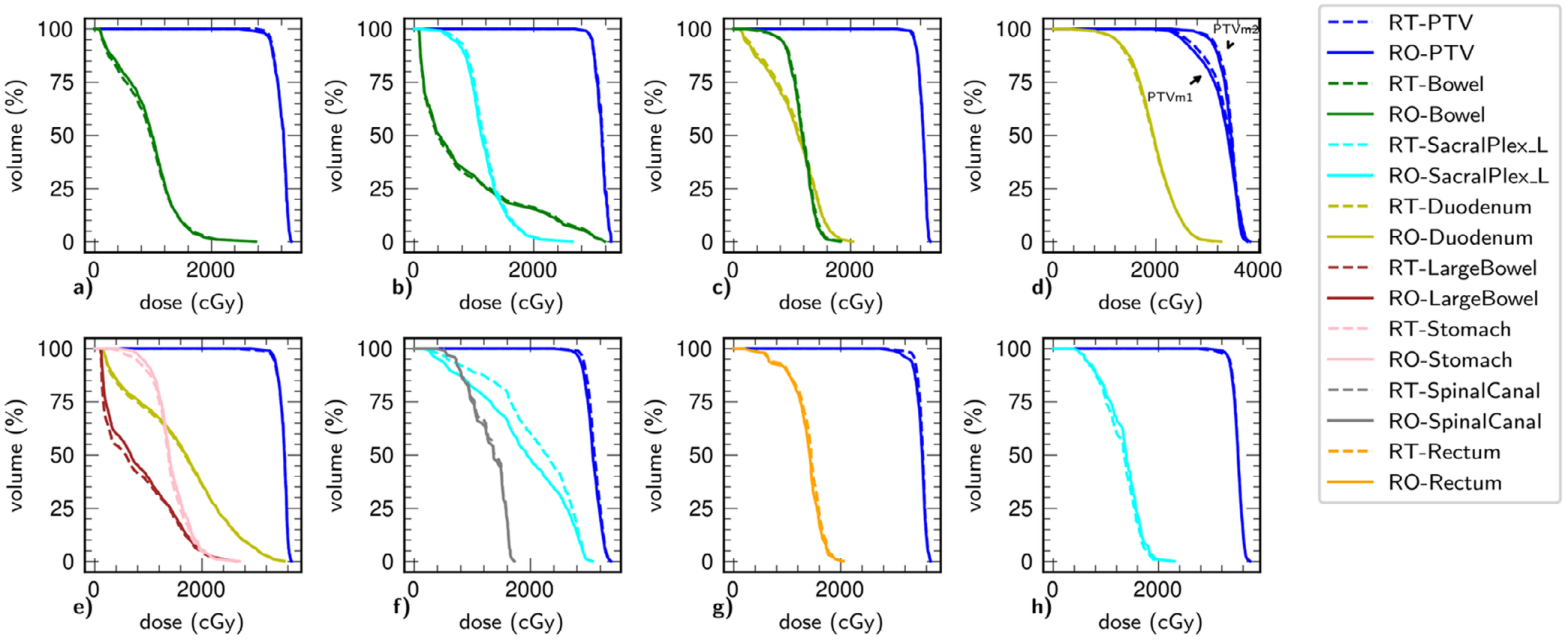
Dose–volume histograms for the target planning target volumes (PTV) in all 8 cases. Simultaneous Truth and Performance Level Estimation (STAPLE) contours derived from radiation oncologist (RO; solid lines) and radiation therapist (RT; dashed lines) are presented. For Case 4, two separate PTVs were present (PTVm1, PTVm2); each were contoured by the RO and RT cohorts respectively.

**FIGURE 5:**
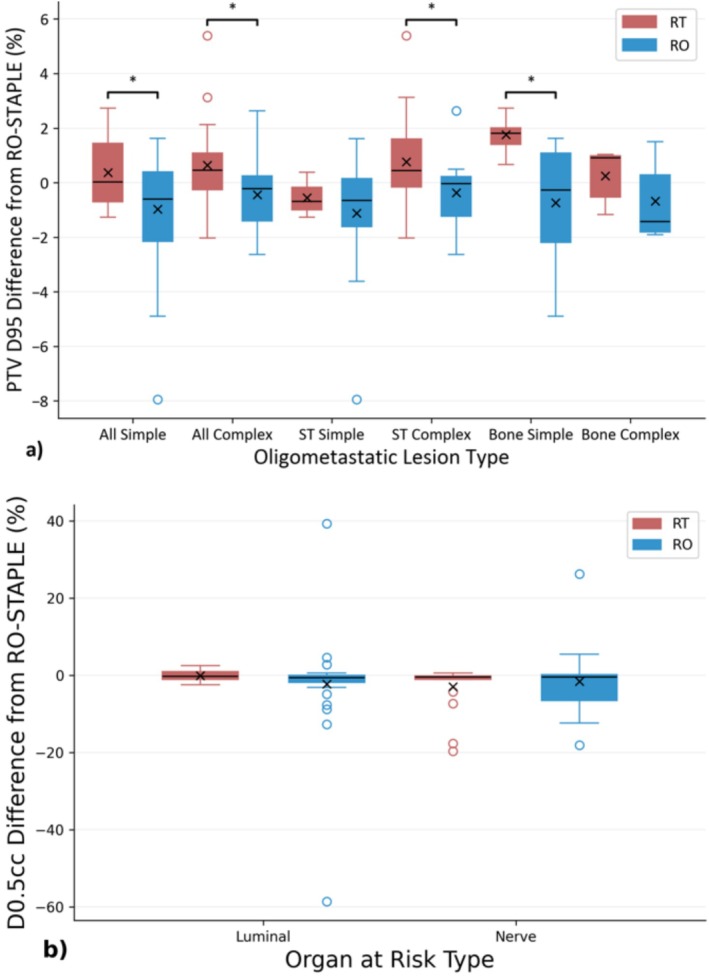
Relative percentage dose difference between radiation oncologist (RO) and radiation therapist (RT) contours and RO consensus Simultaneous Truth and Performance Level Estimation (STAPLE) contour for (a) planning target volume (PTV) dose at 95% volume (D95) and (b) D0.5cc for key organ‐at‐risk volumes in the categories of luminal and nerve structures (full list available in Figure S2). *Categories with *p* < 0.05 for Wilcoxon signed rank test with Benjamini‐Hochberg correction.

**FIGURE 6:**
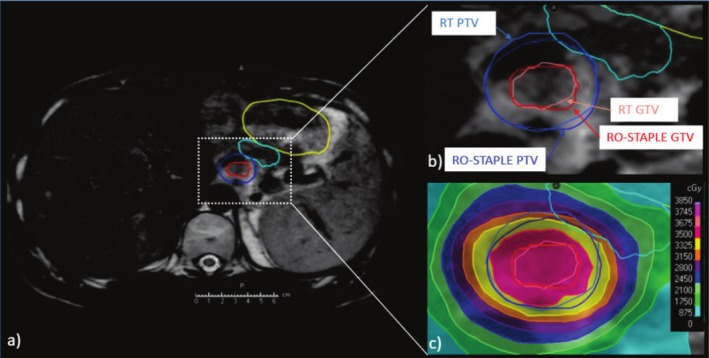
(a) Example MR and contours for a case with > 2% PTV D95 RT difference from the RO consensus Simultaneous Truth and Performance Level Estimation (RO‐STAPLE) contour. As shown in panel (b), small variations between the RT and RO‐STAPLE GTV (red) translate into small PTV (blue) variations. But as shown in (c), these minor differences can have a large impact on maximum dose to a small volume in proximal OARs like the duodenum (cyan) and stomach (yellow).

The PTV and OAR DVHs are illustrated in Figure 4 for all RTT‐ and RO‐generated contours for each patient. Mean difference in PTV D95 coverage compared with the corresponding RO‐STAPLE generated PTV, normalised to the prescribed dose across all 45 generated contours was 0.5% ± 1.5% for RT and −0.7% ± 1.9% for RO cohort, and reached statistical significance (*p* < 0.05). Figure 5a shows the PTV D95 differences from the different target classifications, showing that PTV coverage was significantly greater for RT contours compared with RO contours for all simple and all complex targets, driven by the complex soft tissue and simple bone targets. The PTV D95 dose differences from the RO‐STAPLE exhibited ‘Acceptable deviation’ for 23/45 RT and 20/45 RO contours, exhibited ‘Minor deviation’ for 20/45 RT and 21/45 RO contours, exhibited ‘Clinically relevant deviations’ for 1/45 RT and 3/45 RO contours and exhibited ‘Major deviation’ (dose > 5%) in 1/45 RT and 1/45 RO contours. The major deviation in PTV D95 for the RT contour was +5.4% and was −7.9% for the RO contour. Cases that resulted in a PTV 95 change > ±1% were spread across cases, complexities, and from varying participants. Only one RT contour exhibited PTV D95 difference > 2% for a ‘soft tissue—complex’ case in which GTV qualitative score was > 4 and DICE was a reasonable 0.79, but the PTV overlap with OARs was the greatest creating a steep dose gradient (Figure 6).

We considered dosimetric impact on luminal and nerve OARs separately (full list available in Figure S2). The OAR D0.5cc difference from RO‐STAPLE was −1.1% ± 5.9% for RTs and −1.1% ± 12.0% for ROs averaged for all OARs, with no statistically significant differences for either luminal or nerve OARs (Figure 5b). Outliers observed for nerve D0.5cc were concentrated on sacral plexus contour from a single case for both cohorts while the luminal D0.5cc outliers were found only in the RO cohort (*n* = 8) and were spread across 6 different OARs. Maximum deviation (+39.3% and −58.6%) of luminal D0.5cc was from the RO cohort and resulted in dose differences (2873 and 3209 cGy) below our 3500 cGy institutional clinical goal and therefore would not have influenced plan acceptance.

The mean (±standard deviation) Likert score for each group was 4.8 (±0.4) and 4.7 (±0.5) for RTs and ROs respectively. When scoring the GTV contours all RTs and ROs received a ‘pass’ of 90%. As such none of the RTs required additional feedback or additional contouring cases. Only one case had a GTV which was scored as a ‘fail’ which was a ‘soft tissue complex’ case from the RO cohort. The mean (±standard deviation) Likert score for each group of OAR contours was 4.8 (± 0.5) for RTs and 4.5 (± 0.7) for ROs. All cases where the OARs ‘failed’ were luminal structures with the 5 incidences spread across 4 different cases. Of these 5 instances, 4 came from the RO cohort (pelvic bowel *n* = 2, abdominal bowel *n* = 1 and duodenum *n* = 1) and 1 from a duodenum contour in the RT cohort.

## 4 Discussion

To our knowledge, this is the first comprehensive evaluation of geometric accuracy and dosimetric uncertainty for both target and OARs volumes in MR‐guided ART for OMD. To date, few studies have considered RT‐led ART workflows beyond prostate [9, 10], and we believe the evaluation of OMD is timely given the increasing utilisation of MR‐guided ART. Here we compared both RT and RO contours versus the RO‐STAPLE contour for our geometric and dosimetric evaluation. Previous publications on geometric accuracy of RT contouring in the adaptive space have chosen to use either the contour created offline by a single clinician [10, 15] or the contour generated in the online session [8, 9]. While both options fail to account for inter‐clinician variability, the second option does not account for imperfect contours created under the pressures of the online setting. The use of a consensus contour for GTV and OAR volumes overcomes both limitations.

Given limited published data specifically evaluating MR‐guided OMD contouring variability, our results will provide a key benchmark for future comparisons. One previously published report of 2 RT contoured oligo‐lymph cases found target DICE coefficients of 0.63–0.85 [9], which was slightly lower than our RT (0.68–0.91) and RT‐Staple DICE coefficients (0.80–0.91). The range of mean DICE coefficients for RT contouring of prostate target volumes has been reported as 0.85–0.97 and 0.93–0.96 in the literature [8, 16]. The wider range of DICE coefficients observed in our OMD study compared with previous prostate reports may be related to the range of targets included in our analysis or to the smaller target volumes for OMD as small delineation variations may lead to lower DICE coefficients compared with larger prostate volumes as demonstrated in Figure S4. Furthermore, our dosimetric analysis suggests the GTV delineation differences do not lead to a clinically significant impact on the PTV target coverage.

A key strength of our study is the inclusion of PTV coverage metrics, as our results show that cases deemed a qualitative ‘fail’ or those with low DICE scores do not necessarily translate to a clinically meaningful change in PTV D95. We dosimetrically evaluated all 9 GTVs from the 8 cases for all 10 participants, going beyond only evaluating outliers as previously performed [7]. This ensures that all targets and OARs were successfully contoured and resulted in clinically equivalent dose‐volume metrics for both targets and OARs. Note that greater PTV coverage relative to RO‐STAPLE is not necessarily desired over lower PTV coverage, as increased PTV coverage could relate to systematically smaller GTV contours. This may in fact be the driver of statistically significant differences between cohorts seen in the soft tissue complex and bone simple classifications.

Clinical implementation of the RT‐led workflow may vary by centre. While some centres do not require direct RO involvement [7, 9] for prostate cases, our centre has chosen to model previously published work that requires the RO to provide direct supervision for the first fraction and then be reachable for consultation during subsequent fractions [8, 15]. We have an additional triage for extra‐hepatic, non‐pancreas OMD patients in which the treating RO will categorise cases as eligible or ineligible for a RT‐led workflow either upfront or during the planning process. RT‐led workflow eligibility, such as target visibility and definition and patient‐specific considerations, is then confirmed and documented at the first adaptive treatment session.

Implications of this study may not be directly transferable to other countries or centres. In our study, initial feasibility was achieved with a single anatomy workshop and 8 retrospective training cases compared to other studies [17], but all RTs in our cohort were previously internally credentialed for RT‐led prostate ART treatments and 4/5 have additional MRI training, which could have resulted in a higher‐than‐normal baseline competency. There may also be considerations in countries where dosimetry is not in the scope of practice of all RTs, but instead requires a separate designation. Other barriers may exist, such as in the US payor environment, as the clinician still needs to be paged for final contour review and plan evaluation [18].

Multiple study limitations exist, including the small single institution participant cohort. Contouring performance may be influenced by participants observing the reference contours on reference MRI, but also using the deformably registered OARs as a starting point instead of de novo contouring. Vaassen et al. [19] demonstrated that resultant contours are impacted by existing contours, but starting with mapped contours at each fraction is required for an efficient workflow and relevant for this study. Similarly, the knowledge and skills demonstrated by the RTs to reproduce the daily target contours required for online ART are not the same as the knowledge and experience required to define said volumes for the initial treatment plan. While this means that RTs are more reliant on protocols and training to re‐create contours compared to ROs, this may also be the reason for higher concordance among the RTs, as seen in Figures 4 and 5, as well as other publications [20]. Additionally, we applied a time constraint to simulate the online workflow, which may have impacted both RT and RO contouring performance. Future directions, now that feasibility has been established, would be to implement the next phase of evaluation involving eligible online OMD cases where therapist performance is scored and possible intervention provided by the supervising RO.

## 5 Conclusions

Well trained RTs accurately performed daily online re‐contouring of the target and OAR volumes, resulting in high quality contours when benchmarked against a consensus contour created by ROs in an initial pilot evaluation. Additionally, we did not observe clinically significant dosimetric changes as a result of contour variations. We have demonstrated that an RT‐led online adaptive workflow for OMD is feasible, with contouring accuracy equivalent to ROs that optimises resources to improve advanced radiotherapy technique access and supports future trends of increasing use of local ablative OMD treatments as well as maximising healthcare resources.

## Author Contributions

**Amanda Moreira:** methodology, investigation, writing – original draft, writing – review and editing, data curation, formal analysis, visualization. **Winnie Li:** conceptualization, writing – review and editing, writing – original draft, methodology, supervision. **Iymad R. Mansour:** methodology, investigation, writing – original draft, writing – review and editing, formal analysis, data curation, visualization, software. **Mame Faye:** investigation, writing – review and editing, formal analysis. **Ali Hosni:** writing – review and editing, methodology. **Aruz Mesci:** writing – review and editing. **Enrique Gutierrez‐Valencia:** writing – review and editing. **Patricia Lindsay:** writing – review and editing, methodology, conceptualization. **Peter Chung:** writing – review and editing, methodology, conceptualization. **Jeff Winter:** conceptualization, methodology, investigation, writing – original draft, writing – review and editing, visualization, software, data curation, formal analysis.

## Ethics Statement

This retrospective study was approved by the University Health Network Quality Improvement Review Committee (ID #24‐0793).

## Conflicts of Interest

Ali Hosni: Non‐financial leadership of liver TSG at ELEKTA MRL consortium. The other authors declare no conflicts of interest.

## Supporting information


**Data S1:** ara70016‐sup‐0001‐DataS1.docx.

## Data Availability

The data that support the findings of this study are available from the corresponding author upon reasonable request.
