# Supplementary material for: Feasibility of Therapist‐Driven MR‐Guided Adaptive Radiotherapy for Oligometastatic Disease: Geometric Accuracy and Dosimetric Impact
Source: J Med Imaging Radiat Oncol. 2025 Aug 26;69(8):777–86. doi: 10.1111/1754-9485.70016 (PMC12675315; doi:10.1111/1754-9485.70016)
Supplement: Supplementary file 1 — Data S1: ara70016‐sup‐0001‐DataS1.docx. [file ARA-69-777-s001.docx]

**Supplemental Material**

| **Metastasis** **Location** | **Cases Treated** | **Basis for ineligibility** | **Ineligible** | **Eligible** | **Selected for Study** |
| --- | --- | --- | --- | --- | --- |
| Soft Tissue | 32 | Mix target types in single plan | 2 | 30 | 5 |
| Bone | 9 | Mix target types in single plan | 1 | 9 | 3 |
| Liver | 17 | Target motion | 17 | 0 | 0 |
| Pancreas | 5 | Anatomic variation and target motion | 5 | 0 | 0 |

Supplemental Material Figure 1. Table of inclusion and exclusion criteria for phase 1 of our training program: initial feasibility assessment of RTT contouring for OMD. Patients on clinical trials with planning constraints outside the departmental standard were excluded to ensure standard dosimetric evaluation.


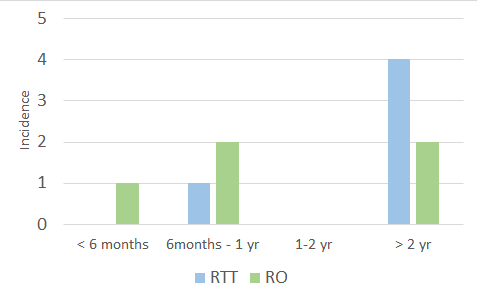

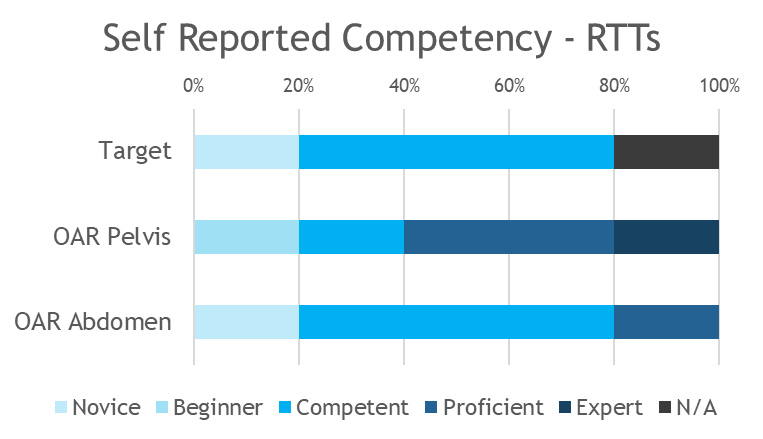

Supplemental Material Figure 2. Survey results given to participants showing years of MRL specific experience and self-reported competency for various ART tasks prior to training program.

| Case | Luminal OARs | Nerve OARs |
| --- | --- | --- |
| Case 1 | Bowel | N/A |
| Case 2 | Bowel | Left Sacral Plexus  Right Sacral Plexus |
| Case 3 | Duodenum  Bowel | N/A |
| Case 4 | Duodenum | N/A |
| Case 5 | Duodenum  Large Bowel  Stomach | N/A |
| Case 6 | N/A | Left Sacral Plexus  Spinal Canal |
| Case 7 | Rectum | N/A |
| Case 8 | N/A | Left Sacral Plexus |

Supplemental Material Figure 3. List of OAR structures included in luminal and nerve categories per case.


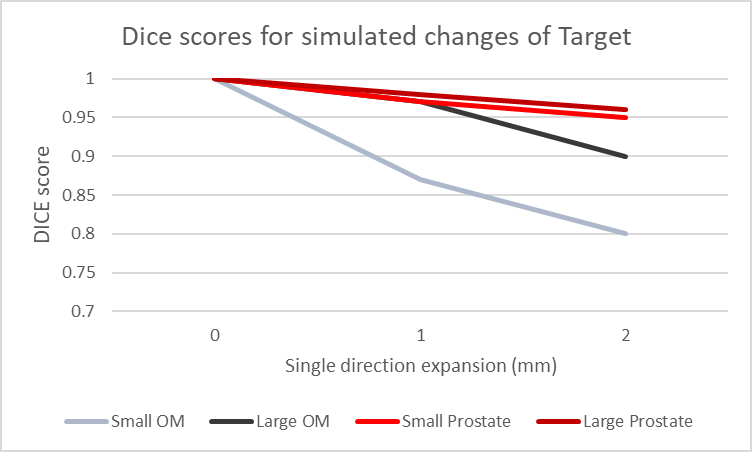


Supplemental Material Figure 4. Influence of target volume on DICE calculations for various Oligometastatic (OM, grey) and Prostate (red) target sizes.  OM volumes taken from study population where Small OM GTV = 0.2cc and Large OM = 15.1cc. Prostate volumes taken to represent intuitional averages, where Small = 50cc and Large = 80cc.
